# Prevention of Transfusion-Associated Graft-versus-Host Disease by Irradiation: Technical Aspect of a New Ferrous Sulphate Dosimetric System

**DOI:** 10.1371/journal.pone.0065334

**Published:** 2013-06-07

**Authors:** Lucas Sacchini Del Lama, Evamberto Garcia de Góes, Paulo César Dias Petchevist, Edson Lara Moretto, José Carlos Borges, Dimas Tadeu Covas, Adelaide de Almeida

**Affiliations:** 1 Physics Department, School of Philosophy, Sciences and Letters of Ribeirão Preto, University of São Paulo (FFCLRP/USP), Ribeirão Preto, São Paulo, Brazil; 2 Mathematics, Statistics and Physics Institute, Federal University of Rio Grande (IMEF/FURG), Rio Grande, Rio Grande do Sul, Brazil; 3 Regional Blood Center of Ribeirão Preto, Ribeirão Preto, São Paulo, Brazil; 4 Center for Cell-Based Therapy, School of Medicine of Ribeirão Preto, University of São Paulo, Ribeirão Preto, São Paulo, Brazil; Beth Israel Deaconess Medical Center, Harvard Medical School, United States of America

## Abstract

Irradiation of whole blood and blood components before transfusion is currently the only accepted method to prevent Transfusion-Associated Graft-Versus-Host-Disease (TA-GVHD). However, choosing the appropriate technique to determine the dosimetric parameters associated with blood irradiation remains an issue. We propose a dosimetric system based on the standard Fricke Xylenol Gel (FXG) dosimeter and an appropriate phantom. The modified dosimeter was previously calibrated using a ^60^Co teletherapy unit and its validation was accomplished with a ^137^Cs blood irradiator. An ionization chamber, standard FXG, radiochromic film and thermoluminescent dosimeters (TLDs) were used as reference dosimeters to determine the dose response and dose rate of the ^60^Co unit. The dose distributions in a blood irradiator were determined with the modified FXG, the radiochromic film, and measurements by TLD dosimeters. A linear response for absorbed doses up to 54 Gy was obtained with our system. Additionally, the dose rate uncertainties carried out with gel dosimetry were lower than 5% and differences lower than 4% were noted when the absorbed dose responses were compared with ionization chamber, film and TLDs.

## Introduction

Transfusion-Associated Graft-Versus-Host Disease (TA-GVHD) is a rare, but fatal potential complication that occurs when viable donor T lymphocytes proliferate and engraft in susceptible patients after transfusion [1]–[3]. At least three factors appear to be directly related to the risk of TA-GVHD [4]: 1) the susceptibility of the patient immune system to the engraftment, 2) the degree of Human Leukocyte Antigen (HLA) similarity between donor and recipient and 3) the number and viability of donor T lymphocytes present in the transfused components.

According to the literature, cases of TA-GVHD have been reported in severely immunocompromised patients, including patients with congenital immunodeficiencies, in bone marrow transplant recipients, as well as in cancer patients treated with chemotherapy or radiotherapy [2], [5]–[15]. This serious transfusion associated adverse reaction has also been reported in presumed immunocompetent patients who received blood from homozygous donors with shared HLA haplotypes or from a family member [16]–[35].

Although the minimum concentration of lymphocytes in the donor blood that can initiate TA-GVHD is unknown, a number of lymphocytes as low as 1×10^4^/kg of the recipient weight may be sufficient to cause TA-GVHD [36]. The referred report corroborates with other works and indicates that even leukocyte-depleted blood products can promote this reaction [37], [38]. Because there is no effective treatment for TA-GVHD [39], [40], irradiation of whole blood and blood components prior to transfusion is the only proven method to prevent the reaction [41], [42]. Ionizing radiation breaks the DNA molecules of T lymphocytes and prevents an immune response against the recipient [3], [43], [44].

Blood irradiation can be performed using commercial irradiators specifically designed for this purpose, which are usually located in blood banks. These dedicated blood irradiators use radioactive isotopes such as ^137^Cs or ^60^Co, which emit gamma-rays, or linear accelerators, which emit X-rays. Based on previous data about the elimination of allogeneic reactivity using Mixed Lymphocyte Culture analysis (MLC), at least 15 Gy was recommended for irradiation of blood components [45], [46]. However, at least three cases of TA-GVHD were reported in patients who received blood irradiated with doses between 15 and 20 Gy [47]–[49]. More recently, studies on the radiosensitivity of T-cells to gamma and X-rays, assessed by Limiting Dilution Analysis (LDA), have shown that an absorbed dose of 25 Gy is necessary to prevent TA-GVHD [28], [44], [50]. Under this exposure condition, damages are minimal to granulocytes and anucleate cells as erythrocytes (RBCs) and platelets (PLTs). Thus, Food and Drug Administration (FDA) [51] and the American Association of Blood Banks (AABB) [52] specify a dose of 25 Gy at the middle plane of the blood component. Similarly, the European and the British guidelines state that a minimum dose should be 25 Gy and no more than 50 Gy for each blood bag [53]–[56]. In order to meet these requirements, a dosimetric system must determine 1) the dose rate and the dose in the blood bags and 2) the spatial dose distribution in the irradiated blood volume.

A Fricke dosimeter is a chemical dosimeter, first proposed in 1927 by Hugo Fricke and Sterne Morse [57] as an acidic oxygenated ferrous sulphate solution. The absorbed dose is inferred through the radiation induced oxidation process, in which ferrous ions (Fe^+2^) oxidize to ferric ones (Fe^+3^) due to water decomposition [58], [59]. This dosimeter has been recommended by the American Association of Physicists in Medicine (AAPM) [60] and the International Commission on Radiation Units and Measurements (ICRU) [61] as an alternative clinical dosimeter owing to its water-equivalent radiological characteristics and achievable absoluteness.

At the end of the last century, many researchers proposed tissue-equivalent dosimeters based on the original Fricke liquid solution, some with gelatin and polymeric matrices instead of liquid. Gelatin dosimetry has been studied since 1950’s [62], [63], when the gel molecular proprieties began to be studied after ionizing radiation exposures. Some of these gelatins were used to prepare Fricke gel dosimeters, becoming the first dosimetric system able to three-dimensionally map the absorbed dose in a non-destructive and non-invasive manner [64]–[66]. In fact, one of the most common recipes to prepare this type of dosimeter adds porcine skin gelatin and Xylenol Orange dye to the original solution. This dosimeter is known as Fricke Xylenol Gel (FXG) and was proposed by Gillboy *et al* in 2000 [67]. The FXG has a linear absorbance response for doses from 0 up to 30−40 Gy. It has advantages over the liquid solution when spatial resolution and natural oxidation stability are crucial. In addition, the FXG dosimeter allows quantitative and qualitative analysis of the absorbed dose spatial distribution over an irradiated volume [68], [69] with various analysis techniques, including UV/visible spectroscopy [67], [70]–[75], magnetic resonance [64]–[66], [71], [76]–[80], Charge-Coupled Device (CCD) [69], [81]–[90] and photoacoustics [91], [92].

Because the standard FXG optical response saturates for absorbed doses higher than 30−40 Gy, we have developed a modified recipe for the FXG dosimeter, which includes lowering the pH, increasing the metal dye concentration and adding sodium chloride to the solution. These modifications provide linear responses for doses up to 50 Gy, as recommended by the blood irradiation guidelines.

## Materials and Methods

### Irradiation Sources and Calibration

In this study, the standard FXG [67], TLDs (LiF-100, Harshaw Chemical Co., Ohio, USA), radiochromic film (Gafchromic-EBT2, International Specialty Products, New Jersey, USA) and the modified FXG dosimeter were irradiated using one sealed ^60^Co source (effective energy of 1.25 MeV) from a cobalt teletherapy unit (Theratron-780C, MDS Nordion, Ontario, Canada). Calibration of the ^60^Co source was accomplished with a calibrated clinical ionization chamber (Farmer-N30001, PTW, Freiburg, Germany) and an electrometer (K35617EBS, Keithley Instruments Inc., Ohio, USA), in accordance with the International Atomic Energy Agency (IAEA) protocol recommendations [93]. Also following this protocol, the standard FXG, TLDs and film were calibrated in terms of absorbed dose in water and their responses were compared with those obtained with the modified FXG dosimeter. In this sense, the standard FXG, TLDs and film were employed as reference dosimeters to validate the modified FXG response for dosimetry of a gamma blood irradiator (Gammacell^®^ 3000, Best Theratronics Ltd., Ontario, Canada). This irradiator contained one sealed ^137^Cs source (effective energy of 0.662 MeV) with nominal activity of 53.7 TBq (1,450 Ci), inside of a steel-encased lead shield, able to deliver up to 5.0 Gy per minute at the canister central plane, for default rotation rates (approximately 30 cycles per minute). The blood irradiator dosimetry setup consisted of two different radiation phantoms proposed here.

Since blood and blood components are usually chilled or frozen, standard and modified FXG dosimeters were used for different irradiation temperatures, employing the ^60^Co teletherapy unit and a water phantom, maintained at the desired temperature (3 and 23±1°C were evaluated). FXG samples were located inside 1.0×1.0×4.5 cm^3^ acrylic cuvettes (Plastibrand™, Sigma-Aldrich, Missouri, USA), which were positioned 0.5 cm under the water surface, in accordance with IAEA build-up procedures [93]. The dosimeters were isolated from water by a thin PVC plastic film, in order to avoid possible contamination.

### FXG Dosimeter System

The modified FXG recipe was prepared considering 124.38 mM of porcine skin gelatin (270 Bloom, Gelnex, Santa Catarina, Brazil), 0.63 mM of ferrous ammonium sulphate hexahydrate (Merck, Darmstadt, Germany), 0.20 mM of Xylenol Orange, XO, (Merck, Darmstadt, Germany), 48.78 mM of sulphuric acid (J.T. Baker, New Jersey, USA), 0.63 mM of sodium chloride (Sigma-Aldrich, Missouri, USA) and Milli-Q water (Millipore, Massachusetts, USA). Owing to its convenient melting point (40°C, approximately) and visible light transparency, porcine skin gelatin was chosen as the matrix gel. Ferrous ammonium sulphate is the crucial component of the FXG dosimeter because its oxidation (transformation from Fe^+2^ ions to Fe^+3^ ones) is proportional to the absorbed dose in the irradiated dosimeter. These chemical changes produce a visible light spectral band due to XO, a metal ion salt indicator, which bonds only to the Fe^+3^ ions. The FXG induced absorbance after a radiation exposure allows the use of optical techniques, such as spectrophotometry and the CCD system described here. In order to prevent immediate aggregation between XO and naturally oxidized Fe^+3^ ions, sulphuric acid is necessary to reduce the solution pH. Sodium chloride was added to the modified solution to minimize influences from organic impurities and to increase the system reproducibility.

In this research, we studied solutions with pH between 1.0 and 2.5 and XO concentrations between 0.05 and 0.25 mM, in order to reach an adequate FXG recipe for blood irradiation, which is stated hereafter. Considering the modified FXG dosimeter and 100.0 ml of Milli-Q water, dissolve 5.0058 g of gelatin and 0.0037 g of sodium chloride in 75.0 ml of water. Heat the mixture up to 45°C and continuously stir until a clear solution is obtained. Then, dissolve 0.0152 g of XO in 26.0 ml of sulphuric acid and add 12.5 ml of water to this solution. Finally, dissolve 0.0196 g of ferrous ammonium sulphate hexahydrate in the remaining 12.5 ml of water and add the last two solutions to the warm gelatin one. After thorough mixing, fill the appropriate cuvettes for the desired application and cool to obtain a consistent gelatin.

Analysis depends on the fact that, after ionizing radiation exposure, the XO molecules bond 1∶1 with Fe^+3^ ions, which result in dyed regions with Optical Densities (*OD*s) directly proportional to the absorbed dose at every point in the volume. The pre- and post-irradiation images were registered with a CCD image system using a 24 mm focal length, a f/18 aperture and a shutter speed of 1/100 [86]–[90]. Later, both images were superimposed and the *OD* variations were processed by a Matlab ^®^ (MathWorks, Massachusetts, USA) computational routine, considering the red and green channels for the modified FXG and film, respectively. We used the following expression:
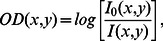
(1)where *OD(x,y)* is the optical density of the pixel at (*x*, *y*), i.e., the *OD* of the *x^th^* line crossing the *y^th^* column of the acquired image. The quantities *I*_0_ *(x,y)* and *I(x,y)* are the color levels of the (*x^th^*, *y^th^*) pixel corresponding to the pre- and post-irradiation samples, respectively. Thereby, it was possible to establish a relationship between the absorbed dose and the generated *OD* for any radiochromic dosimeters, such as FXG and film. Additionally, a spectrophotometer (Ultrospec*^TM^* 6300, General Electric Co., Buckinghamshire, England) was used to compare the absorbance responses between the standard and the modified FXG dosimeters, selecting the 585 nm spectral band and computing the quantity:
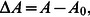
(2)in which 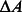 is the absorbance difference between irradiated, A, and non-irradiated, A0, samples.

### Dosimetry of the Blood Irradiator

Two-dimensional maps, i.e., the absorbed dose spatial distributions, were determined considering a completely filled canister with blood phantoms composed of water or appropriate plastic materials, such as polystyrene or acrylic [41], [45], [94]. The dosimeters and their corresponding phantoms were positioned inside the blood irradiator canister and irradiated for 1.0 minute. Analysis used the mirrored middle plane dose distributions of the canister, which were plotted as isodose curves.

**Figure 1:**
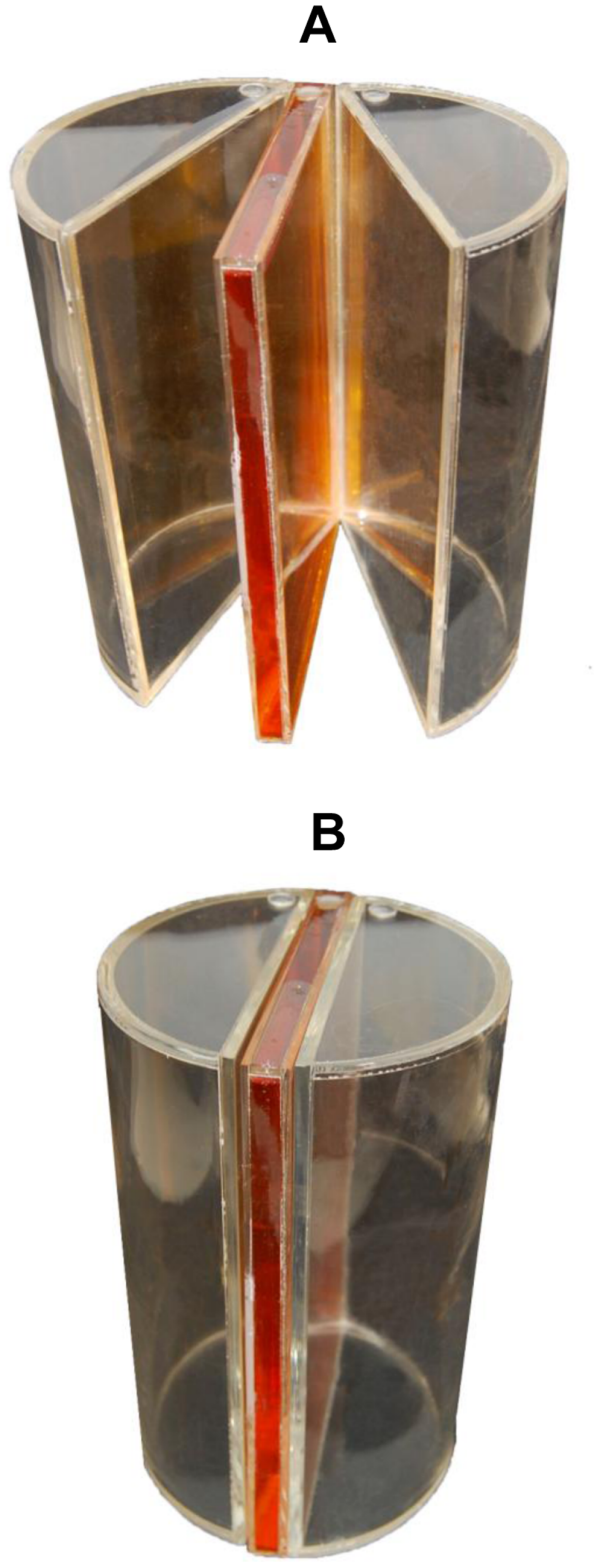
The FXG dosimetric phantom assembly used in the blood irradiator. (A) shows a central cubic dosimeter cuvette filled with the dosimeter and surrounded by two semi-cylindrical water phantoms. (B) shows the mounted FXG phantom.

A dedicated FXG phantom was constructed for the blood irradiator dosimetry. In this phantom, the FXG dosimeter was placed in a cubic cuvette (12.4×19.4×1.0 cm^3^), surrounded by two semi-cylindrical cuvettes filled with water, as shown in Figures 1A and B. These cuvettes were manufactured with 2.0 mm-thick walls for adequate ^137^Cs build-up purposes [95]. The phantom geometry was chosen to match that of the canister and simultaneously allow acquisition of the absorbed dose distributions in the central middle plane of the volume.

The same acrylic phantom employed with the FXG dosimeter (Figure 1) was used to perform the blood irradiator dosimetry with film. Thus, a 12.4×19.4 cm^2^ film sheet was inserted between the cubic and one of the semi-cylindrical water filled cuvettes. Film determined absorbed dose distributions were evaluated considering the same parameters as those applied for FXG dosimetry, i.e., absorbed doses were determined by the Optical Density method described earlier and mirrored isodose curves were used to represent dose distributions at the central middle plane of the phantom.

**Figure 2:**
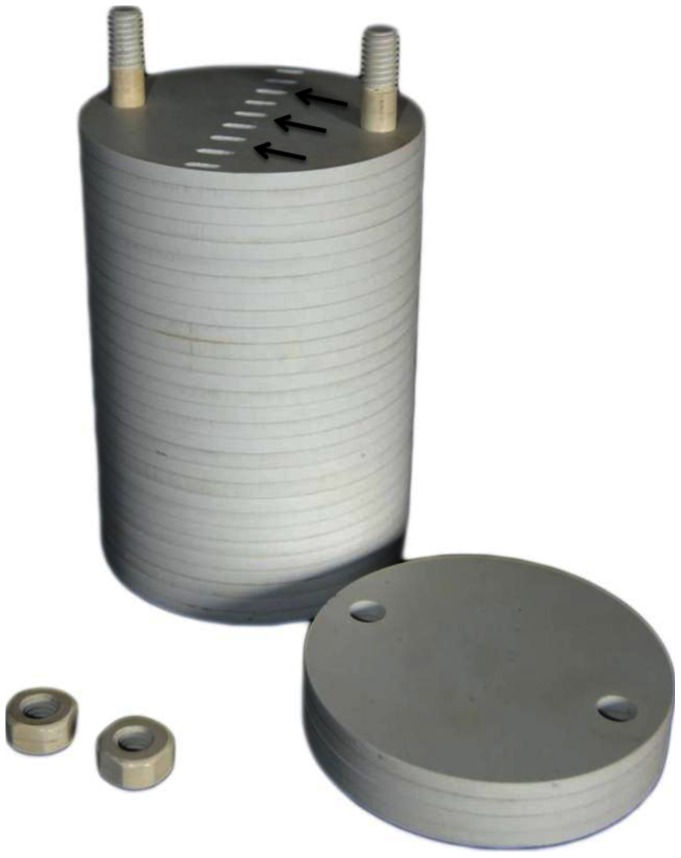
Clear polystyrene phantom employed in blood irradiation thermoluminescence dosimetry (TLD). Arrows show some cavities where TLD dosimeters were allocated.

A cylindrical homogeneous clear polystyrene phantom was also manufactured, with size and shape matching those of the canister, to measure absorbed doses with TLDs. Clear polystyrene plaques were cut in accordance with the canister dimensions and attached face to face with screws of the same material. Absorbed doses along the central plane of the phantom were measured using three TLDs per cavity, diametrically distributed along some plates (Figure 2). After aligning the plates, the TLD-loaded phantom was positioned inside the blood irradiator canister and then irradiated for 1.0 minute. TLDs were previously annealed according to the manufacturer recommendations, i.e., 1 h at 400°C followed by 2 h at 100°C. Dose readings were obtained with a Harshaw reader (2000-B/2000-C, Thermo Fisher Scientific Inc., Massachusetts, USA) and a cubic spline interpolation technique was used to represent dose distribution along the central plane of the phantom.

### Statistical Analysis

In our study, we considered ten replicated batches for each delivered dose, which ranged from 0 up to 54 Gy. In order to represent the results obtained here, statistical analyses were performed considering averages and standard deviations. The combined standard uncertainty was calculated according to the method stated in the Guide to the Expression of Uncertainty in Measurements [96] and in the ISO/EASTM Estimating Uncertainty in Dosimetry for Radiation Processing [97].

## Results

### Calibration

Dosimetry of the ^60^Co beam was performed inside a water tank with an ionization chamber using a 10×10 cm^2^ field size, at 80 cm source-to-dosimeter surface distance [93]. The ionization chamber yielded a dose rate of 0.95 Gy per minute, with an uncertainty of less than 3%. For the same irradiation setup, the dose rate measurements were 0.94 Gy per minute with TLD, 0.93 Gy per minute with film and 0.91 Gy per minute with the standard FXG, all uncertainties lower than 5%. The dose rate measured with our modified FXG was 0.97 Gy per minute, with an uncertainty of 4%.

### Dose Response and Relative Sensitivity of the Modified FXG Dosimeter

**Figure 3:**
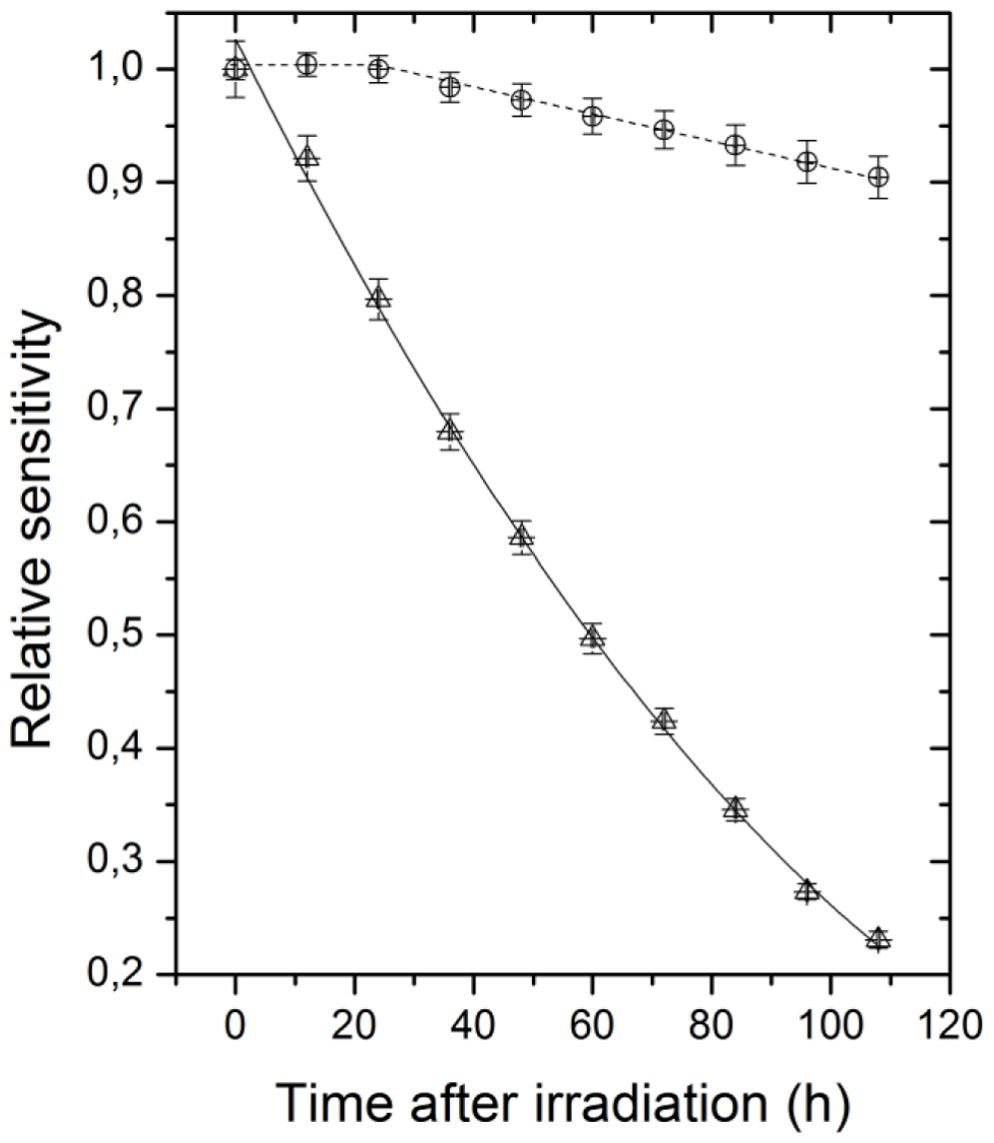
Relative sensitivities for the standard (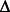) and for the modified (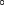) FXG dosimeters *versus* time after irradiation. Irradiations were performed considering a known ^60^Co therapy field.

In a dark and temperature controlled (5±1°C) environment, absorbances were found to fade exponentially with time after irradiation for the standard FXG, while the modified dosimeter showed no significant fading effects in the first 24 h (Figure 3). After the first day of irradiation, the modified FXG dosimeter showed a smooth linear fading, which persisted during five days of storage.

**Figure 4:**
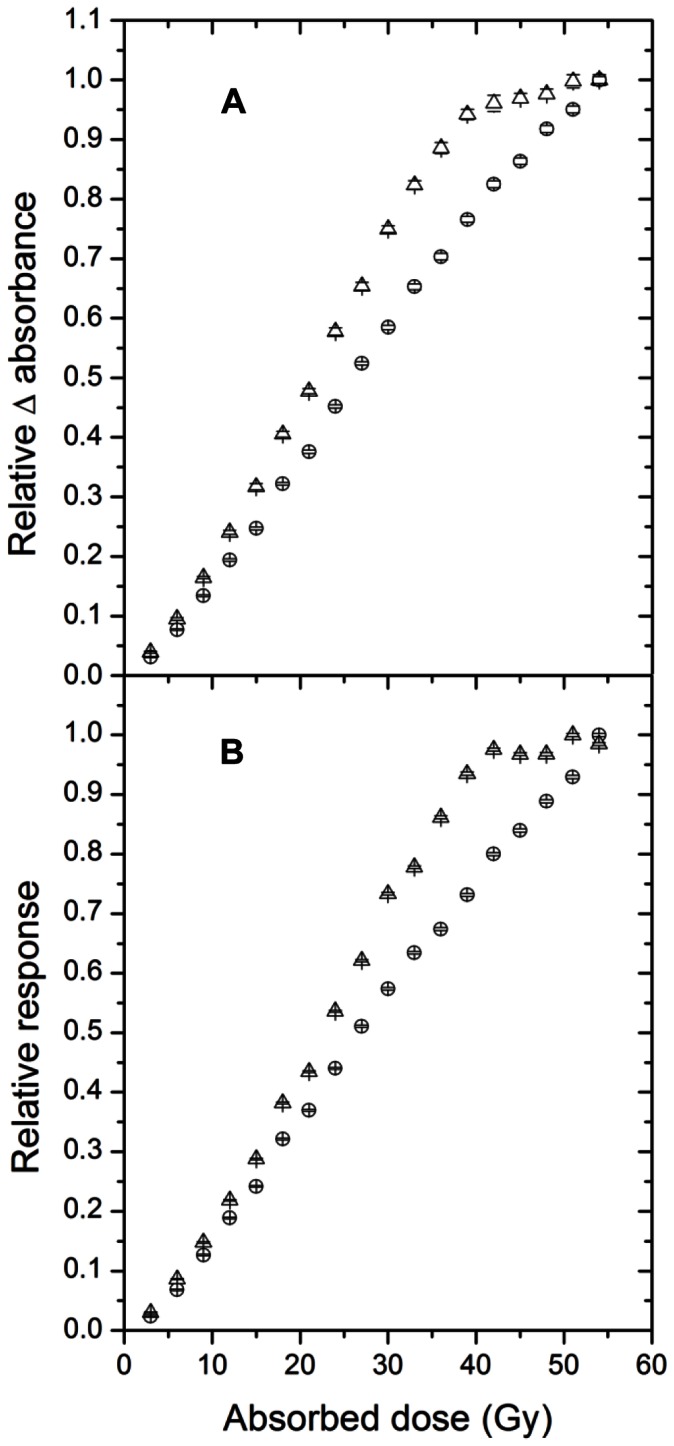
Calibration curves obtained for the standard (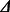) and the modified (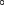) FXG dosimeters. (A) presents the spectrophotometer absorbance variations, while (B) presents the CCD *OD*s.

Figure 4A presents the standard and the modified FXG determined absorbed doses up to 54 Gy, using a spectrophotometer to select the 585 nm absorbance band. The standard dosimeter saturated for doses higher than 40 Gy, while our modified FXG was linear through the full dose range, with linear correlation coefficient (r^2^) better than 0.995. An absorbed dose resolution of 0.2 Gy was attained with this setup. Figure 4B presents the *OD* responses for the CCD optical-based technique, compared with those measured by the spectrophotometer. The results are in agreement with those acquired by the previous technique, i.e., the standard FXG dosimeter saturates for higher absorbed doses, while the modified one is linear over the whole range (r^2^ = 0.997). For this analysis technique, a maximum absorbed dose resolution of 0.5 Gy was attained.

The standard FXG dosimeter showed a sensitivity variation of 0.13±0.01% per °C for the spectrophotometric data and of 0.21±0.02% per °C for the CCD measurements. In contrast, for the same temperature range, we observed no significant dependencies for the modified FXG dosimeter.

### Dosimetry of the Blood Irradiator Unit

The modified FXG and the film dosimeters were used in combination with the water phantoms (Figure 1) and measured by the *OD* method, while TLDs were used in combination with the clear polystyrene phantom (Figure 2) and measured by the Harshaw reader. The ^137^Cs blood irradiator central dose rates measured with the modified FXG, film and TLDs dosimeters resulted in 5.41, 5.49, and 5.41 Gy per minute, respectively. The dose rate uncertainties associated with TLD and film dosimeters were lower than 3.5%, while the modified FXG dosimeter presented an uncertainty lower than 4.5%.

**Figure 5:**
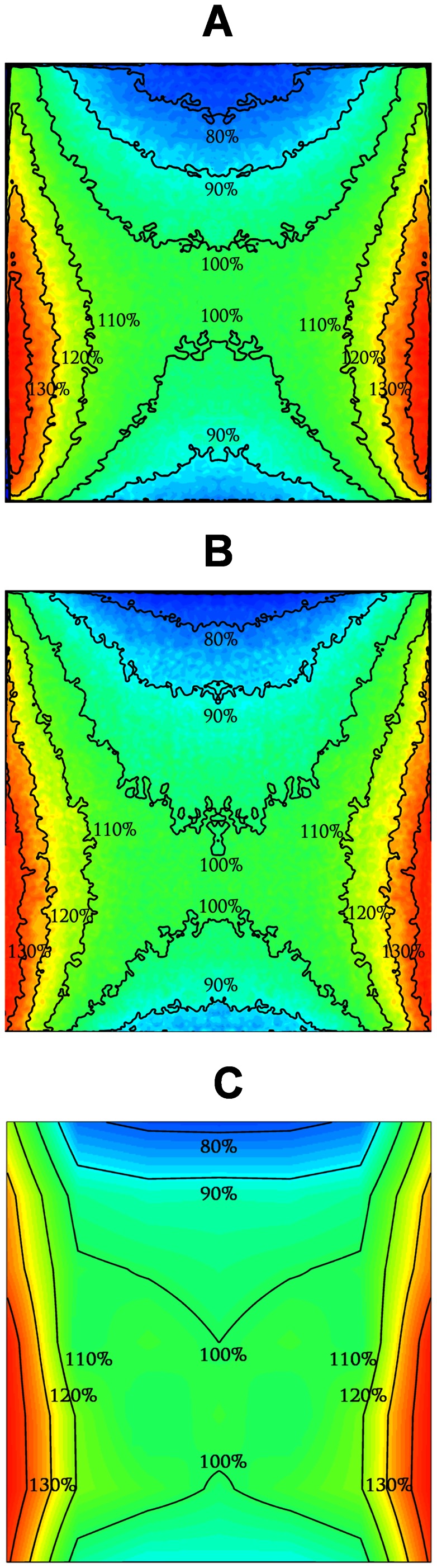
Isodose curves inferred at the canister central plane. Irradiations were performed at the blood irradiator considering (A) the modified FXG,(B) the film and (C) the TLD dosimeters.

Figure 5 presents the dose distributions at the central middle plane of the phantoms, in 10% step isodose curves and a color scale. The modified FXG (Figure 5A), the film (Figure 5B) and the TLDs (Figure 5C), show absorbed doses ranging from 74.2±3.7% up to 138.3±4.4%, from 73.6±1.1% to 139.3±1.4% and from 77.7±0.9% to 137.9±2.8%, respectively.

## Discussion

Ionization chambers present high accuracy (<3%) and are employed as reference dosimeters by many protocols [60], [93], [98], [99]. For this reason, we used an ionization chamber as a reference dosimeter to calibrate the modified FXG dosimeter proposed here. According to the literature, film and LiF-100 dosimeters also present appropriate features for dosimetric purposes [100]–[103]. For these reasons, we also selected these dosimeters to validate the modified FXG dosimeter when the blood irradiator was used. Although the standard FXG can also be used for this purpose, saturation effects for doses higher than 40 Gy are the main limitations for its use for blood irradiation. As seen in the calibration results, the dose and dose rate values obtained by the modified FXG dosimeter and those obtained by the ionization chamber were not significantly different. In the same manner, the dose rate associated with the ^137^Cs blood irradiator determined by the modified FXG dosimeter was not significantly different than those obtained by the standard FXG, film and TLD dosimeters.

FXG is a spectrophotometrically feasible gel matrix [67], which allows optical-based techniques to be applied for data acquisition. Beyond that, the FXG dosimeter response presents low dependence for energies up to 1.25 MeV [104], which avoids the correction factors necessary when this dependence is not negligible. Additionally, the porcine skin gelatin preserves the spatial absorbed dose distribution integrity of the irradiated sample and reduces ions diffusion after exposure. Furthermore, this gelatin is chosen as the gelling agent because it helps to maintain a low FXG effective atomic number (3.35 at 1 MeV) [105] and an adequate mass density (1.05 g per cm^3^), which are similar to those of whole blood (3.45 and 1.06 g per cm^3^, respectively) [106], [107]. Lastly, this gelatin also provides two important features, a reasonable melting point (40°C, approximately), which facilitates preparation, and lower costs than other polymers. For all these reasons, we have developed this dosimeter to optimize its sensitivity, stability and linearity in dose response over the entire dose range used in blood irradiation, besides proposing an appropriate phantom for dosimetric measurements.

According to our findings, we observed a well stabilized relation between the FXG dose response and its pH (non-published data). The pH controls the chemical equilibrium of water radiolysis and consequently the rate associated with the production of free radicals. A higher pH value (>2.0), i.e., more alkaline solution, promotes premature Fe^+2^ oxidation, which leads to early saturations of the FXG response (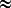
30−40 Gy) and reduction on its linearity (r^2^<0.985). In contrast, a lower pH (<1.8) seems to decrease the free radicals production rate and to increase the linear dose response range. This feature is desirable since it allows determination of doses as high as 50 Gy, currently recommended by the guidelines [51]–[56]. This low pH range also results in higher values of linear correlation factors associated to the FXG response (r^2^>0.995), which allows one to acquire information from the response-curve in an effortless way. However, for very low pH values (<1.3), XO−Fe^+3^ can dissociate and data related to dose may be lost. Porcine gelatin structure appeared not to be affected by the pH values investigated (1.0 up to 2.5).

Another important issue concerning the FXG dosimeter is the relation between the solution acidity and its sensitivity. Considering the pH range used here, we observed that the sensitivity associated with the solution was maintained constant (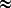
0.070 Gy^−1^cm^−1^) for pH values higher than 2.0, while it decreased when the pH was reduced. Due to oxygen atoms dissolved in the FXG solution, ferrous ions, Fe^+2^, are naturally oxidized into ferric ones, Fe^+3^, which diffuse through the solution. Both effects can be reduced when the solution pH is lowered, although they continue to occur with time. We observed that a pH value of 1.6 presents reduced natural oxidation and diffusion effects. For these reasons, we propose a FXG recipe with a pH value of 1.6.

We noted that higher Xylenol Orange concentrations yielded wide linear dose ranges and decreased diffusion effects. As stated earlier, XO bond to Fe^+3^ ions in a 1∶1 ratio. Although the standard FXG recipe presents a ferrous concentration able to provide responses for doses as high as 100 Gy, its XO concentration does not obey the referred ratio. In fact, similar to the dosimeter pH, the standard XO concentration also contributes to the FXG saturation effects for doses at 30−40 Gy. For a 0.20 mM XO concentration, we obtained adequate linearity for doses up to 54 Gy and reported no significant sensitivity dependence. In this sense, considering the FXG optical response and its corresponding linearity, we propose a FXG recipe with a XO concentration of 0.20 mM.

Sodium chloride aims to control the radiolysis chain reaction, which is initiated in the solution by ionizing radiation. This occurs since Cl atoms prefer to oxidize ferrous ions rather than producing peroxides that accelerate the radiolysis process. We observed better FXG repeatability responses (<3.5%) for a 0.63 mM sodium chloride concentration and lower fading effects when compared to the standard FXG dosimeter. Adequate repeatability and fading are important features for any dosimetric instrument that is intended to provide reliable measurements in a quality control program.

According to our study, the standard FXG response showed a dependence on temperature of 0.13±0.01% per °C, for 3 up to 23±1°C, in agreement (<8%) with results available in literature [108]–[110]. On the other hand, dependence on temperature showed by the modified FXG dosimeter was not significant. Therefore, the modified FXG is a preferred dosimeter when longer times, such as those for teletherapy blood irradiations (>30 min), are required.

Usually, FXG samples are analyzed at 585 nm in a spectrophotometer. In this study, we used a CCD system in order to better investigate the proprieties related to the FXG dosimeter. The CCD system used in this study allowed us to use three different primary reading channels, namely Red (R), Green (G) and Blue (B). We observed that the red and the green channels presented optimum readings for the FXG and film dosimeters, respectively, when linearity was considered, allied with acceptable sensitivity responses. According to the results obtained here, the CCD resolution in dose was almost 50% lower than that of the spectrophotometer one. However, the linearity responses for both techniques were similar (r^2^ = 0.995 for the spectrophotometer and r^2^ = 0.997 for the CCD system). While the spectrophotometer is recommended when a higher accuracy in dose is desirable, the CCD system provided adequate linear responses for the FXG dosimeters, as well as high spatial resolutions (<0.5 mm) [90], being completely acceptable for blood dosimetry purposes.

Although blood irradiation has been suggested since 1970s [111] and associated quality control for blood products is well established among different agencies [51]–[53], [55], [56], limited attention has been given to the dosimetric aspects of this practice. Qualitatively, there are blood irradiation indicators, used routinely, which only state whether or not the blood bag was irradiated. Different quantitative dosimetric tools have been studied for blood dosimetry in recent years: thermoluminescent dosimetry (TLD) [94], [112], [113], Fricke solution [112], radiochromic film [114], colorimetric dosimetry [115] and solid state dosimetry, including methacrylate polymers (*red perspex*) [94], alanine [94], [116]–[118], mosfet [41] and diodes [119]. A quantitative method is commercially available for blood irradiators dosimetry (Dose-Map ^TM^/Ashland Inc., Covington, Kentucky, USA). However, data need to be mailed to the manufacturer for dosimetric results, not at all desirable for quality control.

The blood dosimetric methods employed used phantoms made of acrylic, water and clear polystyrene (Figures 1 and 2), easily found materials. Simultaneously, they provide similar mass attenuation coefficients to those of whole blood in the appropriate radiation energy range (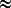
2.0%; <1.0% and <2.5%, respectively) [120], in accordance with ionizing radiation protocols [121]. Moreover, acrylic and clear polystyrene do not need highly specialized equipment to be machined and are relatively inexpensive. In fact, the proposed dosimetric phantoms can be readily employed in any blood bank quality assurance program.

The isodose curves obtained with FXG, film and TLD dosimeters were similar (Figure 5). The maximum difference in dose observed among the dosimeters was 4%. Despite spatial resolutions of these dosimeters are slightly different, those differences are not relevant in comparison to the minimum and maximum recommended doses. Although the TLD normalized absorbed dose distribution was similar to the other dosimeters, its spatial resolution was lower, because TLD dosimetry is commonly accomplished through individual TLD dosimeters, meaning that discrete readings need to be acquired and later interpolated. Each dosimeter presented reasonable homogeneous regions at the central irradiation area, due to continuous rotation of the canister, and a highest isodose percentage values at the lower mid point of the irradiated volume. Those dose values were presumed to be related both to photon scattering and source alignment. Since the canister top is without a cap, irradiations were not vertically symmetric. However, the energy imparted by the ^137^Cs photons to the phantom was relatively low (0.662 MeV) and indicates that scattering was not the major contributor to the reported results. Thus, data suggest that the ^137^Cs source or, at least, the radioactive volume of the source may be misaligned from the vertical center. Nevertheless, even if the available blood irradiator presents a displaced source, the FXG, film and TLD percentage dose distributions indicate that it is still able to irradiate blood at acceptable homogeneity levels.

As recommended by different international guides [51]–[56], blood is currently irradiated from 25 up to 50 Gy to prevent TA-GVHD. According to our findings, the modified FXG dosimeter provides feasible and linear responses for blood irradiation applications in this absorbed dose range. In addition to dose rate and dose distribution measurements, the proposed FXG dosimetric system can be used by the blood bank staff for a quality assurance method for blood irradiation. In summary, it has been shown that the modified FXG dosimetric system proposed here presents appropriate features for quality assurance control in the clinical environment.
