# Correction: Prevention of Transfusion-Associated Graft-versus-Host Disease by Irradiation: Technical Aspect of a New Ferrous Sulphate Dosimetric System

**DOI:** 10.1371/annotation/a0305338-b6a2-44a9-b154-02b2e128395c

**Published:** 2014-01-03

**Authors:** Lucas Sacchini Del Lama, Evamberto Garcia de Góes, Paulo César Dias Petchevist, Edson Lara Moretto, José Carlos Borges, Dimas Tadeu Covas, Adelaide de Almeida

In the "FXG dosimeter" subsection of the "Materials and Methods" section, second paragraph, fourth sentence,"... 0.0152 g of XO in 26.0 ml of sulphuric acid..." should read "... 0.0152 g of XO in 0.26 ml of sulphuric acid ..."

